# Digital Medical Device Companion (MyIUS) for New Users of Intrauterine Systems: App Development Study

**DOI:** 10.2196/24633

**Published:** 2021-07-13

**Authors:** Toeresin Karakoyun, Hans-Peter Podhaisky, Ann-Kathrin Frenz, Gabriele Schuhmann-Giampieri, Thais Ushikusa, Daniel Schröder, Michal Zvolanek, Agnaldo Lopes Da Silva Filho

**Affiliations:** 1 eHealth and Medical Software Solutions Bayer AG Wuppertal Germany; 2 Regulatory Medical Device Excellence Bayer AG Berlin Germany; 3 Medical Affairs Statistics Bayer AG Wuppertal Germany; 4 Medical Affairs, Real World Evidence and Outcomes Data Generation Bayer AG Berlin Germany; 5 Medical Affairs Bayer SA Sao Paulo Brazil; 6 BAYOOMED Medical Software Development BAYOONET AG Darmstadt Germany; 7 Medical Affairs Bayer AG Berlin Germany; 8 Department of Gynecology and Obstetrics Federal University of Minas Gerais Belo Horizonte Brazil

**Keywords:** medical device, levonorgestrel-releasing intrauterine system, mobile medical app, mobile phone

## Abstract

**Background:**

Women choosing a levonorgestrel-releasing intrauterine system may experience changes in their menstrual bleeding pattern during the first months following placement.

**Objective:**

Although health care professionals (HCPs) can provide counseling, no method of providing individualized information on the expected bleeding pattern or continued support is currently available for women experiencing postplacement bleeding changes. We aim to develop a mobile phone–based medical app (MyIUS) to meet this need and provide a digital companion to women after the placement of the intrauterine system.

**Methods:**

The MyIUS app is classified as a medical device and uses an artificial intelligence–based bleeding pattern prediction algorithm to estimate a woman’s future bleeding pattern in terms of intensity and regularity. We developed the app with the help of a multidisciplinary team by using a robust and high-quality design process in the context of a constantly evolving regulatory landscape. The development framework consisted of a phased approach including ideation, feasibility and concept finalization, product development, and product deployment or localization stages.

**Results:**

The MyIUS app was considered useful by HCPs and easy to use by women who were consulted during the development process. Following the launch of the sustainable app in selected pilot countries, performance metrics will be gathered to facilitate further technical and feature updates and enhancements. A real-world performance study will also be conducted to allow us to upgrade the app in accordance with the new European Commission Medical Device legislation and to validate the bleeding pattern prediction algorithm in a real-world setting.

**Conclusions:**

By providing a meaningful estimation of bleeding patterns and allowing an individualized approach to counseling and discussions about contraceptive method choice, the MyIUS app offers a useful tool that may benefit both women and HCPs. Further work is needed to validate the performance of the prediction algorithm and MyIUS app in a real-world setting.

## Introduction

### Background

The importance of digital health and the role of software in clinical care are well recognized [1]. In women’s health care, interactive digital tools are becoming increasingly accepted in terms of supporting health care choices and facilitating discussions between women and health care professionals (HCPs) [2]. Furthermore, the popularity of menstrual cycle tracking apps continues to rise, and at present, there are more than 100 *period tracking* apps available, with downloads surpassing 200 million globally, since 2016 [3,4]. Women use these apps for a variety of reasons, including to become more aware of their bodies, to understand how the body reacts at different stages of the menstrual cycle, to be prepared (for the start of menstruation), and to facilitate conversations with their HCP [5].

Globally, over 922 million women of reproductive age rely on some form of contraception [6]. A woman’s choice of contraceptive method can depend on a variety of factors, including ease and convenience of use, perceived efficacy, associated costs, and expectations of bleeding pattern [7,8]. Many contraceptive methods, such as oral contraceptive pills, implants, levonorgestrel-releasing intrauterine systems (LNG-IUSs), and copper intrauterine devices are associated with alterations in menstrual bleeding patterns [9-11], and some women cite the potential for unfavorable bleeding patterns (such as frequent or prolonged bleeding) or a fear of menstrual irregularity as key reasons for not choosing particular methods [12-15]. Although LNG-IUSs are associated with reductions in menstrual bleeding over time, it is important to note that in the first 3 months following LNG-IUS placement, bleeding and spotting may increase as a result of the local effect of levonorgestrel on the endometrium, which some women may find unfavorable [16-19]. For women deciding to use LNG-IUSs, the desire for less bleeding or amenorrhea (absence of bleeding) is commonly cited as a key reason for choosing this method [19]; therefore, any bleeding or spotting following placement of the LNG-IUS may be perceived negatively and result in dissatisfaction with the method or concern that there is an underlying issue. This could lead to repeat consultations with their HCP to address concerns or, in some cases, the discontinuation of the method [20-24].

Providing thorough contraceptive counseling can help reduce fear and uncertainty and may encourage women to try a method [25]. Counseling should be tailored to the needs of the individual woman, address concerns and preferences, and provide reassurance regarding potential side effects or complications, as well as allow the woman to set realistic expectations regarding her potential bleeding pattern [26-28]. Each woman’s experience of menstrual bleeding is highly individual; in addition, information offered during counseling may not be fully processed, and expectations may not align with the impact that bleeding and spotting changes can have on day-to-day activities. Indeed, although some studies have shown a beneficial effect of anticipatory counseling on method satisfaction and reductions in discontinuation due to bleeding disturbances [29], it has been suggested that counseling interventions with multiple points of contact may further improve adherence and acceptability of contraceptive methods [30].

Available data from clinical trials provide a valuable source of information on bleeding patterns experienced by women using different LNG-IUSs that can be used to aid method selection in clinical practice and can also be used to provide guidance to women on the bleeding pattern they may expect when initiating an LNG-IUS [18-20,31-34]. However, the experience of bleeding following placement is unique to each woman and may be influenced by a variety of factors. Although information on the most commonly expected bleeding patterns from clinical trials helps to convey the general likelihood of having a certain pattern, there is currently no means available to further tailor this information to the individual woman, and help inform her of her personal expected duration or intensity of bleeding.

A tool that can predict a woman’s menstrual bleeding pattern and provide a clinically meaningful output, such as expected duration and intensity of bleeding after device placement, could therefore be a valuable addition to support counseling. Furthermore, after the initial counseling visit and placement of an LNG-IUS, a woman may find that her bleeding pattern is unfavorable and interferes with her quality of life, leading her to seek additional support and reassurance from her HCP to help her manage the situation. Providing an interactive digital app could therefore allow further information to be provided to women in the postplacement period, empowering them to better understand their LNG-IUS and any associated alterations in menstrual bleeding patterns. This may improve confidence and satisfaction with the contraceptive method and encourage continuation, as well as facilitate easier communication between women and their HCPs.

### Rationale

Data gathered through the analysis of daily bleeding diaries used during phase II and phase III clinical trials of LNG-IUS 12 (Kyleena, Bayer AG) indicated that in the initial 90 days following placement of LNG-IUS 12, the most commonly reported menstrual bleeding patterns were prolonged bleeding and irregular bleeding [31,32]. This period of prolonged or irregular bleeding following placement may be perceived as a deterrent to some women when deciding on the contraceptive method or could lead to dissatisfaction and potentially early discontinuation for women using the IUS. Over time, the number of women reporting unfavorable bleeding patterns markedly decreases, with more favorable patterns such as infrequent bleeding and amenorrhea being the most commonly reported patterns at the end of year 1, with menstrual bleeding becoming progressively lighter over the 5-year duration of use [19,32].

It was perceived that providing additional support and information regarding expected future bleeding patterns to women during the initial postplacement interval could provide reassurance and encourage method persistence. Therefore, an algorithm was developed that allowed the meaningful estimation of future bleeding patterns based on evidence collected on an individual basis through a digital daily bleeding diary [34]. The algorithm represents the first tool of its kind to provide an individualized prediction of future bleeding patterns and could be useful to both women and HCPs.

The artificial intelligence (AI)–based algorithm uses 90-day bleeding diary information to predict future bleeding patterns, including intensity and regularity. After the woman has entered 90 consecutive days of menstrual bleeding information into a daily bleeding diary, a random forest approach is applied to assign her expected bleeding intensity pattern into 1 of 3 categories: predominantly amenorrhea (<5% spotting days or <1% bleeding days within days 91-270 [equivalent to ≤8 spotting days or ≤1 bleeding day]); predominantly spotting (women not belonging to the predominantly amenorrhea cluster and with <5% bleeding days [≤8 bleeding days] within days 91-270); predominantly bleeding (all other women, ie, ≥5% bleeding days [>8 bleeding days] within days 91-270). A logistic regression model is then used to estimate the probability of the woman having a regular cycle. The probability of correct classification using the model is high (>70%), and the generated bleeding categories are considered informative [34].

### Objective

To facilitate the use of the algorithm in routine clinical practice, it is important to generate an interface that allows the input of a woman’s bleeding diary information and combines it with a clear and easy-to-understand report of the algorithm output. Therefore, our aim is to develop a mobile medical app (MyIUS), which integrates the collection of daily menstrual bleeding information, the AI bleeding pattern prediction algorithm, and a report of the predicted bleeding category. The app is intended to support IUS users during the initial postplacement period and provide a prediction of future bleeding profiles, facilitate effective counseling, and encourage users to persist with IUS device use. This paper describes the development of MyIUS as a sustainable app in the context of an evolving regulatory landscape.

## Methods

### Development Approach Context

Mobile medical apps are defined as software that can be executed on a mobile platform, which can be used either as an accessory to a regulated medical device or to transform a mobile platform into a regulated medical device [35]. International guidance for developers of mobile medical apps is available from the World Health Organization and European Commission, and guidance on a national level is available from bodies such as the US Food and Drug Administration and UK National Institute for Health and Care Excellence [36-38]. However, there is currently no comprehensive framework for the development process; guidance varies between countries, authorities, and institutions and addresses differing aspects of app design, from data protection principles and technical considerations through to defining medical purposes [39].

The spread of different sources of guidance across agencies means that it can be challenging to ascertain the most appropriate development approach to ensure compliance with all relevant standards and regulations [39]. Furthermore, apps are often global products; therefore, developers frequently need to navigate complex compliance requirements involving different agencies, regulations, and guidelines. It is also important to consider the language and cultural aspects of the app. In addition to language translation, some countries have different measurement units and different numeric and date and time formats. These aspects should be addressed in any development plan to ensure that the app will be accessible and suitable for use in different countries and is sustainable over time.

### Regulatory Context

The MyIUS app is classified as a medical device, which is defined as an instrument, apparatus, appliance or software for a specific medical purpose or purposes that does not achieve its principal intended action by pharmacological, immunological, or metabolic means [37,40]. The MyIUS software is intended to monitor menstrual bleeding and spotting. This means that under the forthcoming European Commission Medical Device legislation (due to be launched in May 2021), the MyIUS software will be designated, according to Rule 11 of Medical Device Regulation (MDR) EU 2017/745, as a moderate risk or Class IIa medical device [41]. This moderate risk classification corresponds to the software safety Class A of IEC 62304.

In terms of medical device software, the new MDR, EU 2017/745, represents a significant upgrade of the default classification. Although the default classification for software according to previous legislation (Medical Device Directive [MDD] 93/42/EEC) is a Class I medical device that allows a self-declaration process to demonstrate conformity with the medical device legislation (with no notified body approval necessary for the technical file), the new default classification of medical device software as Class IIa in the forthcoming legislation requires an active approval process of a notified body based on a review of a technical file in order to obtain a declaration of conformity (European Conformity mark). To meet the regulatory requirements for this medical device category, it is essential to demonstrate conformity with general safety and performance requirements, to provide clinical evidence, and to apply a documented design-control process based on a certified quality management system.

To address these stricter requirements for software in the new European legislation, following the launch of the MyIUS app, users will be invited to participate in a real-world performance study sponsored by Bayer AG. This will allow the collection of complementary evidence in a real-world setting, beyond the data provided by Bayer’s previous clinical trial program, in order to support the transition from a Class I medical device as described by MDD 93/42/EEC to a Class IIa medical device, as described by MDR EU 2017/745 [37,41,42].

**Figure 1:**
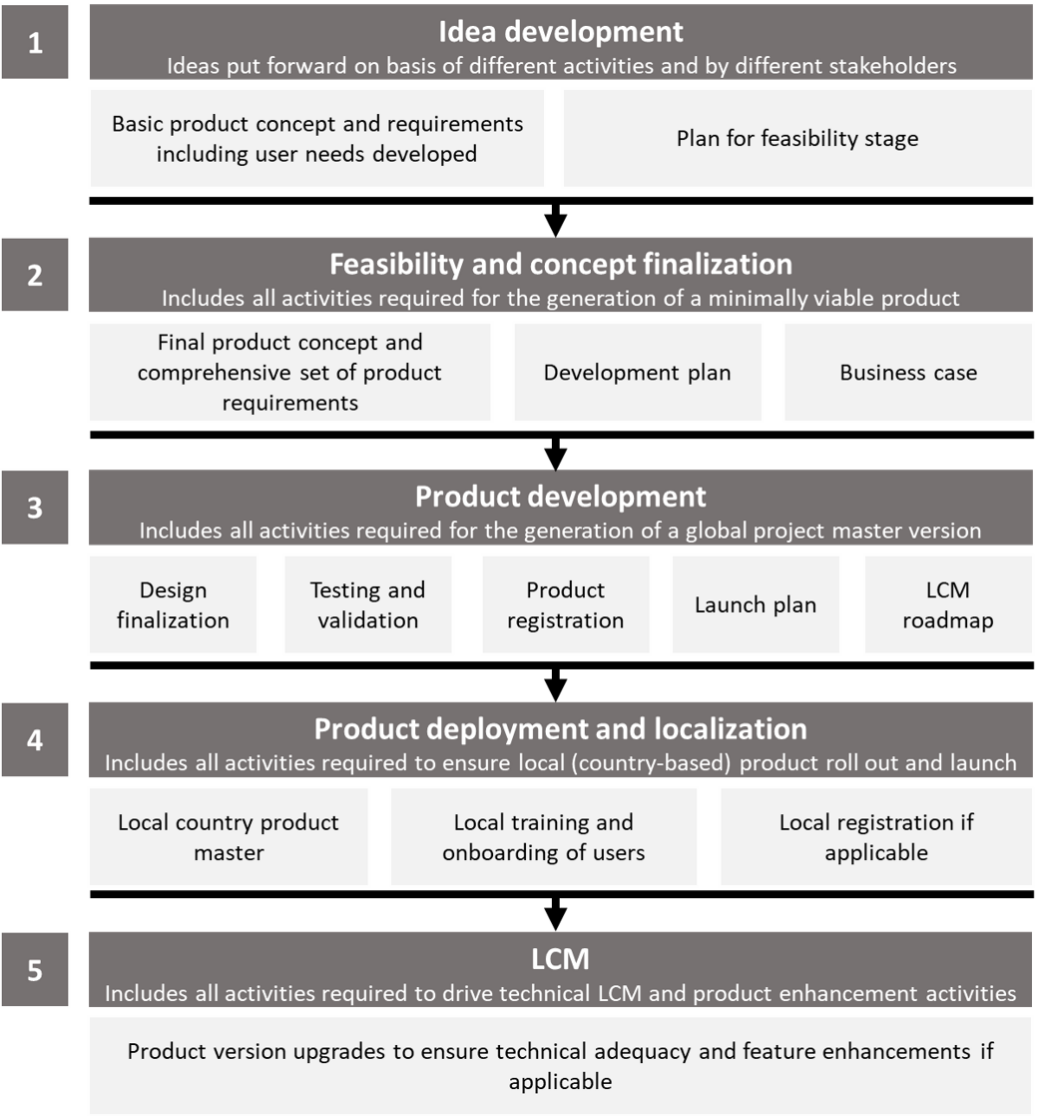
MyIUS app development framework stages. LCM: life cycle management.

The rapidly growing field of medical software apps has led to a constantly evolving regulatory landscape, requiring developers to explore innovative pathways in app design and development. Our medical software product development framework consisted of a phased approach with four key stages, followed by life cycle management comprising a postmarket surveillance plan. The development stages included ideation, feasibility and concept finalization, product development, and product deployment and localization (Figure 1).

### Idea Development

Individual interviews were conducted with a panel of 40 obstetricians and gynecologists in Germany and Brazil to identify the areas of need and inform the development of the MyIUS app. A concept document describing the idea of the MyIUS app was shared with individual panel members. Web-assisted telephone interviews were conducted to gain feedback on the perceived utility and benefits of the app (Textbox 1).

**Perceived benefits and utility of the MyIUS app for health care professionals and intrauterine system users identified during interviews with obstetricians and gynecologists.**

**For health care professionals (HCPs)**

- Could support individualized management and discussion of relevant options with women
- May help HCPs to give more accurate advice to patients about what to expect over the next 3 months
- Patients could be less likely to return to HCPs to discuss bleeding issues, as they know what to expect
- Report tool could provide a realistic view of the woman’s bleeding pattern
- May facilitate more accurate and efficient discussions by reducing reliance on woman’s recollection and subjective description of bleeding

**For intrauterine system (IUS) users**

- IUS users have a personal support tool that they can interact with throughout their postplacement journey
- May help women feel supported and monitored by their HCP throughout the postplacement period
- Digital tool may be better accepted and used by women than a printed leaflet or other such material
- Could improve awareness of bleeding patterns and help to normalize changes after IUS placement
- May increase motivation to continue with IUSs

Insights from these interviews revealed that in routine clinical practice, there is a need for a tool that can support counseling around expected menstrual bleeding changes beyond the initial counseling visit and provide additional information to women during the first 3 to 6 months after placement of the IUS, when they may experience alterations in their normal bleeding pattern. The HCPs interviewed also stated that the tool should be simple to use for easy onboarding and should be self-explanatory, to avoid the need for users to request additional information or explanation from the HCP. For users, there is a perceived need to provide an additional method of support in the initial postplacement period and improve knowledge and understanding of bleeding patterns during this time. The MyIUS app was developed to meet these needs.

The MyIUS app is a collaborative project between Bayer AG (sponsors and developers of the AI algorithm), BAYOOCARE GmbH (legal manufacturers of the app), and BAYOONET AG (ISO 13485–certified software developer of the app) with Concentrix Global Services GmbH providing first- and second-level support. A multidisciplinary team was established to ensure an efficient, high-quality, and compliant design process, which included expertise from the Medical Software Product Lead; eHealth Systems Engineer; Law Patents and Compliance Department; Clinical Development, Medical Affairs, Regulatory Affairs and Clinical Medical Devices divisions; Pharmacovigilance and Pharmaco-device vigilance experts; Medical Software Quality Expert, app development partner, and commercial teams, as well as key insights from the developers of the AI bleeding pattern prediction algorithm. Internal teams from Bayer AG collaborated with external partners from the BAYOOMED division team at BAYOONET AG. Development teams worked in an agile way to provide efficient and robust design and development capabilities.

The key requirements of the MyIUS app identified at this stage were to collect baseline parameters and daily bleeding information for at least 90 days after placement of the IUS, accompany the LNG-IUS user from placement through at least 90 days postplacement offering useful information, and to provide a prediction of bleeding profile for the next 6 months with respect to intensity and regularity based on the collected data, which could facilitate communication between the IUS user and their HCP.

### Feasibility and Concept Finalization

The MyIUS app is intended for users of Bayer’s LNG-IUS (Mirena, Kyleena, and Jaydess or also known as Skyla) following placement. Users may download the app onto their smartphones to monitor and predict the effect of LNG-IUS on menstrual bleeding based on their input of daily bleeding information. The app is not intended to make any diagnostic decision or act as a substitute for evidence-based counseling but instead will act as a *digital companion* or user support tool for women and as a source of bleeding information for HCPs that may facilitate a more individualized counseling approach.

High-level minimum viable stakeholder requirements were defined for the first version; these are presented in Textbox 2. The key stakeholders identified included the end user (main stakeholder), regulatory personnel and regulatory bodies, and quality (both medical and digital) management personnel. A detailed concept and development plan was generated to include all functional and nonfunctional aspects of design, such as verification, validation, usability engineering, and development of the app. These included specifications for stakeholder requirements and software requirements, architecture documentation, module or unit test specification, and software integration test specification. Risk assessment and postmarket surveillance will be conducted after the launch of the app to gain valuable insights into stakeholder requirements and potential issues that may need to be addressed.

**High-level minimum viable user requirements identified for the MyIUS app.**

**App setup and access**

- Unlocking of app with access code
- User to enter baseline data
- Access to frequently asked questions and informative video
- This step should be intuitive and take <15 minutes to complete

**Interaction with the app after placement of the intrauterine system (IUS)**

- Bleeding diary recording bleeding, spotting, or no bleeding
- Reminder function
- Allow backfilling
- Motivational function and gamification (collection of knowledge gems)
- Should include counseling snippets (knowledge gems) for the first 3 months

**Post–90-day interaction with the app**

- IUS user submits data after 90 days
- Result: user assigned to one of three bleeding categories, with description of most likely bleeding pattern
- Reminder to schedule follow-up appointment with health care professional
- Support to motivate user to continue entering bleeding diary data
- PDF copy of bleeding calendar display

**Other**

- Export and import data function (in case of phone switch)
- Frequently asked questions
- Imprint
- User support material
- User manual or instructions for use
- Adverse event reporting via link to appropriate external site

### Product Development

To facilitate integration into the MyIUS app, the original AI bleeding pattern prediction algorithm described by Frenz et al [34] was redeveloped in R 3.6.0 (The R Foundation). Educational content was generated in collaboration with medical affairs team members to ensure the scientific accuracy and validity of the material.

To optimize app design, a usability engineering process was followed in accordance with IEC 62366 [43], which included context analysis, use specification, primary operating functions, and hazardous scenarios (characteristics related to safety and hazard identification). Ongoing iterative testing was performed throughout the development process to optimize the final product. Enhancements identified by usability testing were implemented during software development.

A prototype was developed based on the minimum viable user requirements specified in Textbox 2. Visuals were generated for the setup pages and profile options, menu bar, settings page, support page, legal notice, bleeding report for HCPs, main *home* screen, reminders for when recording of days or recording of bleeding intensity (none, spotting, or bleeding) is missed, calendar, frequently asked questions, prediction day screen (including the report of expected bleeding pattern generated by the algorithm), user feedback page, and educational content pop-ups (so called *knowledge gems*). Within the scope of a formative usability study, the prototype of the app was presented to a panel of 8 women. The panel was considered broadly representative of the envisioned end user of the MyIUS app; women were between the ages of 23 and 48 years and included a mixture of IUS, contraceptive pill, copper intrauterine device, and condom users as well as parous and nulliparous women. All women reported that they would use the app in their daily lives, with most (5/8, 63%) agreeing that the app was “very good” and “user friendly.” Users were asked what they would like to see in the final version of the app and what changes they would make to the existing prototype (Textbox 3). Suggested improvements and desires for the final version were considered for the next stages of development.

**Suggestions for improvement and desires for final version of the MyIUS app collected from the end-user panel.**

**Improvements**

- More colorful design
- Use consistent wording and terminology throughout
- Space for additional notes or information to be entered (eg, exercise)
- Provide more information on the different topics including bleeding and contraception
- Option to mark days without entry as nonbleeding days

**Desires for final version**

- Include additional information about the intrauterine system itself and practical information (eg, how to check threads)
- Ensure app remains easy to access and use, with the same ability to rapidly enter bleeding data
- Reminders to add daily bleeding diary information
- Help section in the app that includes instructions on how to use

Following the completion of the development, verification testing and comprehensive validation testing, including user acceptance testing (summative study), were carried out. These included testing the primary operating functions and hazardous scenarios. A panel of 15 representative end users was engaged virtually (because of restrictions in place as a result of the COVID-19 pandemic) and consisted of women from the United States, Germany, Brazil, and Chile, aged between 20 and 50 years. Women were asked to perform specific tasks and navigate through the app, and their performance was assessed according to predefined criteria (Multimedia Appendix 1). Feedback on app functionality and overall design was also requested from the users (Table 1).

**Figure 2:**
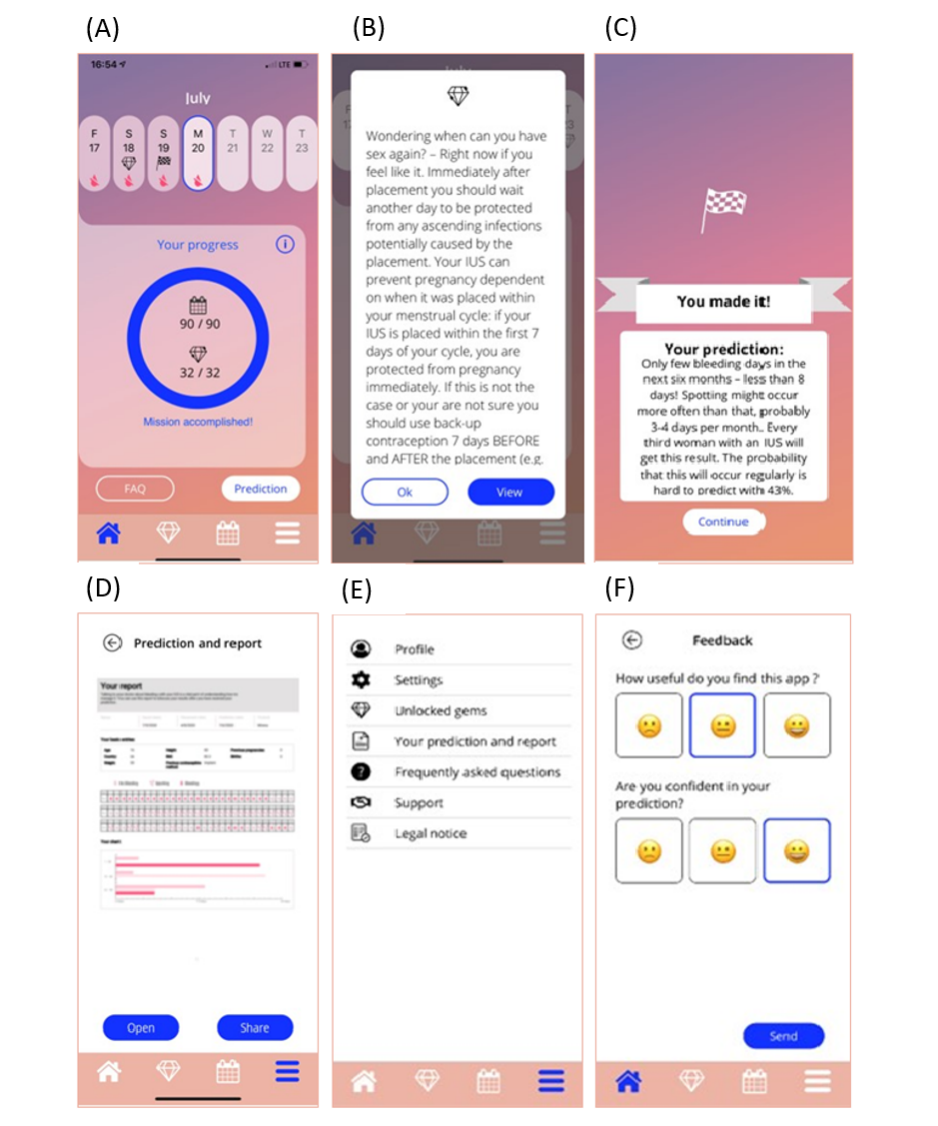
Representative examples of MyIUS app wireframes showing the (A) home screen, (B) pop-up of knowledge gem, (C) bleeding prediction screen, (D) bleeding prediction report for health care professionals, (E) menu screen, and (F) feedback screen.

Representative examples of final wireframes for the MyIUS app, which demonstrate key aspects of app design and highlight the changes that were implemented based on feedback from users, are presented in Figure 2.

Product registration, regulatory submission, and self-certification (according to current MDD 93/42/EEC legislation) were handled by BAYOONET AG.

**Table 1:** Feedback to specific questions regarding MyIUS app in later development stages.

Question	Positive response, n (%)^a^
Would use the app in daily life	13 (93)
Number of setup questions appropriate	11 (79)
Gem concept is clear	11 (79)
Would recommend the app to a friend	10 (71)
Understand the home screen	10 (71)

^a^One woman from the panel did not respond to questions; therefore, positive responses were from a total of 14.

### Product Deployment and Localization

To ensure that the app can be easily localized and upgraded, all text used within the graphical user interface is managed separately from the interface, meaning that the addition of a new language will not require additional programming; instead, a translation of all text will be provided. Local language translation will be performed by a professional translation agency. Transcripts will then be reviewed by legal, medical, and regulatory teams in each country and, in addition, accuracy, colloquial language, and comprehension will be further checked by local language speakers, including an expert HCP. The approved local language transcript will then be implemented by a technical team at BAYOOCARE GmbH.

The app code can incorporate predefined formats (as per device settings) without the need for user interaction. For specific settings where the user might be free to choose within the app, specific conversion logic and rounding are implemented. Local country teams will also ensure compliance of the local country’s product master with the specific data privacy laws of that country.

Local country product masters, local registrations, local training, and local user onboarding have been implemented in specific pilot countries, including Germany (first pilot country), Mexico, France, Spain, Portugal, and the United Kingdom. Launch of the final sustainable app in additional countries will follow.

## Results

### Final Product

The app is available free of charge in the App Store (Apple Inc) and Google Play Store (Google LLC). Once downloaded, the user will need to activate the app using the code supplied by their HCP alongside the LNG-IUS prescription. The global product master has been confirmed to comply with the General Data Protection Regulation to ensure the protection of individual privacy. After a user downloads the app, the data are stored locally on the user’s smartphone. When 90 days of bleeding diary information has been entered, the data are then encrypted and sent as a package to a BAYOONET-managed Amazon Web Services cloud server that runs the AI bleeding pattern prediction algorithm and returns an output to the woman’s device. All data are anonymized and not linked to mobile number, name, or any other personal identifier. No data are stored on the BAYOONET AG server after the algorithm is run.

### Launch and Life Cycle Management

Launch of the MyIUS app in Germany, the first pilot country, took place in early July 2020. The collection of usage metrics, such as the number of downloads, is ongoing and will be used to inform launches in additional pilot countries (such as Mexico, Spain, and Portugal). Following the global launch of the sustainable app, product enhancement activities, including upgrades to ensure technical adequacy and feature enhancements, if applicable, will be performed on a regular basis.

### Real-World Data Collection

To generate complementary evidence to support the upgrade of the MyIUS app to a Class IIa medical device under the new European Commission regulation [41], a real-world performance study is planned. The study will be sponsored and conducted by Bayer AG.

Users of the app will be given the choice to participate in a real-world performance study, and participants will need to provide informed e-consent via the app before enrollment. Participants in the study will use the app to enter 90 days of bleeding diary data and will receive their individual bleeding pattern prediction, and they will then be asked to continue to submit bleeding diary entries for a further 180 days. Data will be gathered in compliance with General Data Protection Regulation; data will be anonymized, and no personal identifiers or mobile numbers will be collected. Data will be saved locally on the user’s smartphone or device, and no data will be stored on the BAYOONET AG server after the algorithm is run.

In addition to generating evidence to allow the upgrade of the MyIUS app, data collected in the performance study will also allow the validation of the AI bleeding pattern prediction algorithm in a real-world setting. The prediction algorithm was developed and trained using data from controlled clinical trials in Kyleena and cross-validated using data from clinical studies of Mirena and Jaydess. The MyIUS app therefore offers an opportunity to gather insights into the performance of the algorithm in the general population and allow additional confirmation of its utility as well as identify further improvements.

## Discussion

### Principal Findings

The MyIUS app was created using a robust and compliant development process that sought insights from HCPs and end users to ensure an app that was of high quality and fit-for-purpose. The end result is an app that will provide a companion to LNG-IUS users in the postplacement period, allowing them to monitor their bleeding pattern and receive an accurate prediction of their expected future duration and intensity of menstrual bleeding.

LNG-IUSs are long-acting contraceptive methods that can be used for 3-5 years. Although some women experience an initial increase in bleeding and spotting during the first 90 days of use, at 12 months, LNG-IUSs are generally associated with a decrease in bleeding days compared with baseline [16-18], although it should be noted that bleeding patterns are considered to be dose dependent and therefore can vary between different LNG-IUSs. More women using the higher dose LNG-IUS 20 (Mirena) experience amenorrhea for example, whereas the users of the lower dose LNG-IUS 8 (Jaydess) report a higher number of bleeding and spotting days than those using LNG-IUS 20 or LNG-IUS 12 [19]. Accordingly, counseling should cover both short-term and long-term bleeding patterns, as well as the benefits, side effects, and risks to help women set realistic expectations [19]. Offering comprehensive counseling on these aspects can improve user satisfaction and continuation with LNG-IUSs [27,29]. However, it is important to note that providing counseling does not completely mitigate the risk of a woman discontinuing with a method as a result of dissatisfaction [44]. Although the discontinuation rate with LNG-IUSs is low overall [33,45,46], discontinuation can have important consequences. Women who discontinue LNG-IUS use may switch to user-dependent or short-acting methods such as condoms, injectables, or oral contraceptive pills or may not switch methods promptly (within 3 months), leaving them at higher risk of unintended pregnancy [44,47,48].

In this regard, MyIUS provides women using IUSs with a means to monitor their bleeding pattern, with the added benefit of generating a meaningful output in the form of a prediction of future bleeding, which could encourage persistence with the method. Through gamification and the provision of knowledge insights, the MyIUS app may help to improve the experience of women in the first months following LNG-IUS placement. The MyIUS app could also provide reassurance to women who experience altered bleeding patterns following LNG-IUS placement and help them feel more in control of the situation. The accurate prediction (probability of correct classification >70%) of future bleeding patterns should also allow women to form realistic expectations about the amount and duration of bleeding they are likely to experience, which can allow them to have better discussions with their HCP regarding their contraceptive method moving forward [34].

Women’s choice of contraception is often influenced by whether HCPs mention or recommend a specific method [49,50]. There are various reasons why HCPs may or may not recommend a specific method, and HCPs’ knowledge of bleeding patterns has been found to be strongly associated with the provision of LNG-IUSs, with those who are unfamiliar with the potential bleeding pattern alterations being less likely to include LNG-IUSs in their counseling [51]. By providing an additional tool to support HCPs with counseling and educate women regarding bleeding patterns, MyIUS could help encourage HCPs to include LNG-IUSs in discussions with women, providing women with a greater choice of contraceptive options that may suit their needs. Reports generated from the MyIUS app may also facilitate discussions of contraception and bleeding patterns between HCPs and women and allow information to be tailored to the individual. Furthermore, tracking apps can also reduce the number of clinical appointments needed and can decrease workloads for HCPs [3].

### Limitations

The MyIUS app was tested by a panel of 23 end users from four different countries (Germany, Brazil, the United States, and Chile) as part of the development process; however, as attitudes, beliefs, digital literacy levels, and personal preferences vary between person to person and country to country, the opinions of the panel may not be reflective of all potential end users. Further insights gathered through the real-world validation study, app store ratings, and user feedback from within the app will be beneficial to confirm the utility of the app in a wider population of users. This will also help inform further updates and improvements to the app in the future.

Initial testing and validation of the AI bleeding pattern prediction algorithm were performed on bleeding diary data from clinical trials of LNG-IUS 12, LNG-IUS 20, and LNG-IUS 8. The planned real-world data study will therefore be essential to collect evidence on the performance of the algorithm in a much wider cohort of women under normal use conditions. Furthermore, the real-world evidence generated will be needed to upgrade the app to a Class IIa medical device under new EU legislation.

Finally, thus far, the AI algorithm and MyIUS app have only been tested and validated in women using LNG-IUSs for contraception. In clinical practice, HCPs may use LNG-IUSs, such as LNG-IUS 20, for other indications such as treating heavy menstrual bleeding; therefore, further investigation is needed to determine the performance of the algorithm and the utility of the app for women using LNG-IUSs for indications in addition to contraception.

### Comparison With Prior Work

The use of personal, digital health informatics is becoming increasingly widespread, and self-tracking of menstruation using apps offers a convenient and easily accessible way for women to manage their own health, allowing them to compare past and average cycles and help identify regularity or irregularity [3,5,52]. The feeling of reward gained by entering data into an app and receiving immediate feedback is suggested to increase motivation and encourage persistence with medical interventions [53]. Features such as gamification and gaining useful knowledge also add to a sense of satisfaction. These aspects may contribute to the popularity of menstrual cycle tracking apps such as Flo (Flo Health Inc), used by over 140 million women worldwide and Clue (Biowink GmbH), used by over 15 million (metrics taken from respective app pages in the App Store and Google Play Store, February 2021).

MyIUS has been developed using a compliant, robust design process involving input from a multidisciplinary team working in an agile way to support the development of a validated mobile medical device. The MyIUS app is the first digital tool designed to support women who choose LNG-IUSs as their method of contraception by using daily menstrual bleeding diary information to provide an accurate prediction of future menstrual bleeding patterns on an individual basis. The app will provide educational insights to users regarding LNG-IUSs and menstrual bleeding patterns and is intended to act as a digital companion for women after placement of an LNG-IUS.

### Conclusions

The MyIUS app has been designed to be simple to use and provides a companion to women during the initial period after IUS placement as well as generating meaningful estimates of bleeding patterns. By tracking menstrual bleeding patterns and estimating expected future patterns, the app may help to facilitate discussions about contraception and bleeding patterns between HCPs and women. The app could also provide reassurance to women who experience altered bleeding patterns following LNG-IUS placement and help them feel more in control of the situation. Furthermore, HCPs may use the information provided by menstrual bleeding diary entries and the AI bleeding pattern prediction algorithm to personalize and enhance counseling about possible bleeding with LNG-IUSs, helping women to set realistic expectations, potentially reducing discontinuation and increasing method satisfaction.

As the desire for customized health care that fits individuals’ unique needs and preferences increases, the MyIUS app offers a first step in making this a reality in the contraceptive counseling and decision-making process for HCPs and women. Further information from user feedback and the planned real-world validation study of the bleeding pattern prediction algorithm are required to inform future refinements and confirm the value of MyIUS for women and HCPs. 

## Appendix

Multimedia Appendix 1 Acceptance criteria and results for usability engineering analysis.

## Abbreviations

AI: artificial intelligence
HCP: health care professional
LNG-IUS: levonorgestrel-releasing intrauterine system
MDD: Medical Device Directive
MDR: Medical Device Regulation
