# Supplementary material for: Digital Medical Device Companion (MyIUS) for New Users of Intrauterine Systems: App Development Study
Source: JMIR Med Inform. 2021 Jul 13;9(7):e24633. doi: 10.2196/24633 (PMC8317031; doi:10.2196/24633)
Supplement: Multimedia Appendix 1 [file medinform_v9i7e24633_app1.docx]

| Use case | Acceptance criteria | Pass/fail |
| --- | --- | --- |
|  |  |  |
| 1 | 90% of the users shall complete the baseline entries questionnaire with the help texts provided by the questionnaire | Pass |
| 2 | 80% of the users shall be able to open the gems section | Pass |
| 3 | 90% of the users shall be able to set bleeding patterns for one or more days | Pass |
| 3 | 80% of the users shall be able to unlock gems | Pass |
| 3 | 90% of the users shall be able to set missing bleeding patterns | Pass |
| 3 | 90% of the users shall be able to recognize missing daily bleeding patterns | Pass |
| 4 | 90% of the users shall be able to set bleeding patterns for one or more days | Pass |
| 4 | 80% of the users shall be able to understand notification contents and follow the workflows the notifications trigger | Pass |
| 4 | 90% of the users shall be able to set missing bleeding patterns | Pass |
| 4 | 90% of the users shall be able to recognize missing daily bleeding patterns | Pass |
| 5 | 90% of the users shall be able to edit daily bleeding patterns already set | Pass |
| 6 | 80% of the users shall be able to edit profile data | Pass |
| 7 | 90% of the users shall be able to receive a prediction | Pass |
| 8 | 80% of the users shall be able to create a PDF report | Pass |
| 9 | 80% of the users shall be able to recognize that baseline parameters are missing before prediction | Pass |
| 9 | 90% of the users shall complete the baseline entries questionnaire with the help texts provided by the questionnaire | Pass |
| 9 | 80% of the users shall be able to understand notification contents and follow the workflows the notifications trigger | Pass |
| Home screen | 80% of the users shall be able to understand the progress area on the home screen | Pass |
